# Wafer scale millimeter-wave integrated circuits based on epitaxial graphene in high data rate communication

**DOI:** 10.1038/srep41828

**Published:** 2017-02-01

**Authors:** Omid Habibpour, Zhongxia Simon He, Wlodek Strupinski, Niklas Rorsman, Herbert Zirath

**Affiliations:** 1 Chalmers University of Technology, Gothenburg 41296, Sweden; 2 Institute of Electronic Materials Technology, Wolczynska 133, 01-919 Warsaw, Poland

## Abstract

In recent years, the demand for high data rate wireless communications has increased dramatically, which requires larger bandwidth to sustain multi-user accessibility and quality of services. This can be achieved at millimeter wave frequencies. Graphene is a promising material for the development of millimeter-wave electronics because of its outstanding electron transport properties. Up to now, due to the lack of high quality material and process technology, the operating frequency of demonstrated circuits has been far below the potential of graphene. Here, we present monolithic integrated circuits based on epitaxial graphene operating at unprecedented high frequencies (80–100 GHz). The demonstrated circuits are capable of encoding/decoding of multi-gigabit-per-second information into/from the amplitude or phase of the carrier signal. The developed fabrication process is scalable to large wafer sizes.

To meet the fast growing demand for telecommunication services, developing high-data-rate communication links in the range of multi-gigabit per second (Gbps) is necessary. The high speed data links can be implemented using either wireless or fiber optic technologies. Wireless technology, particularly in urban areas, has several advantages over fiber optics such as mobility, universal deployment, short installation time and cost effectiveness. However, to achieve data rates comparable to that of the fiber optics, there is a need to develop wireless systems with a very large bandwidth (∼10 GHz). This may be achieved by operating at millimeter wave (mm-wave) frequencies (30–300 GHz)^1^. Even though mm-wave covers a broad range of frequencies, only a certain part of the spectrum is suitable for wireless transmission. This is because atmospheric absorption is only relatively small in these so-called atmospheric windows. The mm-wave atmospheric windows are centered at 35, 90, 140 and 220 GHz^2^. There is therefore a special interest in the 90 GHz band since it simultaneously offers a low loss medium and a large band width (up to 30 GHz). Hence, development of electronic circuits operating in this band is a huge step forward for the realization of multi-Gbps wireless links.

In this regard, graphene is a promising material for the development of mm-wave electronics due to its excellent electron transport properties. Recently, there is a rapid progress in the development of graphene field effect transistors (G-FETs). G-FETs with intrinsic current-gain cutoff frequencies (f_T_) of 400 GHz and maximum oscillation frequency (f_MAX_) of 100 GHz have been demonstrated^3^,^4^. In addition, many G-FET based circuits including frequency multipliers^5^,^6^,^7^,^8^, mixers^6^,^9^,^10^,^11^,^12^, amplifiers^13^,^14^,^15^,^16^ and power detectors^17^,^18^,^19^,^20^ have been presented. Most of the demonstrated circuits so far are not integrated circuits (ICs) requiring external circuitries for operation. ICs allow for high frequency and complex circuits but at the cost of laborious fabrication process. At mm-wave frequencies, broadband circuits can practically only be realized in IC technology. Up to now, there are only few demonstrations of graphene based ICs performing complex wireless communication functions such as signal modulation and demodulation (encoding/decoding information into/from a carrier signal)^21^,^22^,^23^,^24^. The operating frequency of the presented ICs is mainly restricted to a few GHz which is far below the potential of graphene. In addition, the demonstrated data rate is limited to tens of megabits per seconds (Mbps) which is too low for high data rate communications. To compete with existing technologies in high frequency applications, graphene films should exhibit a high carrier mobility as well as a low sheet resistance. These properties can be found in hydrogen intercalated epitaxial graphene on silicon carbide (SiC) substrate^25^. Even though processing ICs on SiC substrate is very challenging, it paves the way for the realization of graphene based high speed data communications.

Here, we present results on monolithic mm-wave IC (MMIC) based on epitaxial graphene in high data rate applications in the 90 GHz band. The developed process is scalable up to full wafer sizes. Currently the limiting factor is not the wafer size but the uniformity of available epitaxial graphene resulting in about 70% yield. The fabricated MMIC has different circuits elements capable of receiving and retrieving information embedded in the amplitude and phase of the carrier signal at the rate of 4 Gbps. Furthermore, the developed circuits are highly linear allowing to generate modulated signal up to the rate of 8 Gbps at 90 GHz band with a bit-error-rate (BER) below 10^−5^. The operating frequency is about 20 times higher and the achieved data rate is more than 200 times better than the previously reported graphene based IC^24^. This work elevates graphene based radio frequency (RF) ICs’ performance to the level that start competing with the existing matured technologies.

## Results

### Epitaxial graphene

Typically, the term “epitaxial graphene” refers to graphene grown on SiC which is in fact not done by epitaxy but by sublimation of silicon. In this study graphene is grown by traditional Chemical Vapor Deposition (CVD) epitaxy using carbon precursor or more accurately by Vapor Phase Epitaxy (VPE)^25^. This method enables the growth of carbon layers directly on SiC surface on both silicon (Si) and carbon (C) polarities with the precision of synthesizing a pre-defined number of carbon layers. The CVD graphene has been studied for both Si-terminated and C-terminated SiC, however, more attention is directed to Si-face growth due to its higher accuracy. To reduce the substrate effect on the synthesized graphene, hydrogen atoms are intercalated and consequently quasi-free-standing (QFS) graphene^26^ is formed. QFS-graphene exhibits much higher and temperature independent carrier mobility, which is desirable for high-speed electronics. The carrier mobility and sheet resistance in our material are 3500–6500 cm^2^/V and 150–250 Ω/Vs respectively. More details of graphene growth and its properties can be found in the method and Supplementary information sections.

### Graphene based high data rate wireless communication landscape

**Figure 1:**
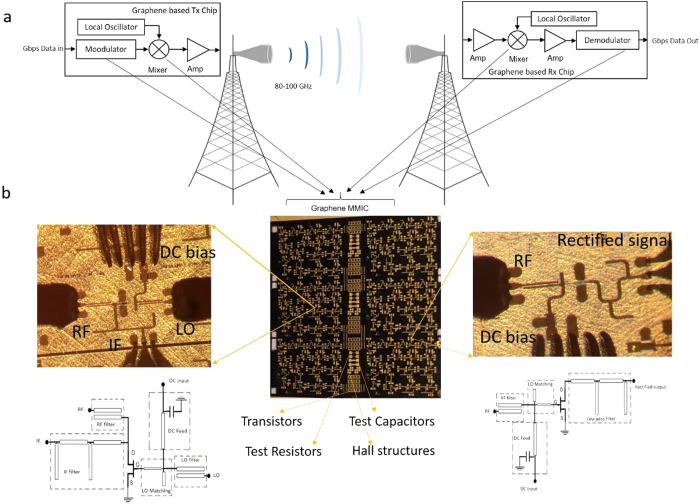
Graphene based high data rate communication landscape. (**a**) A perspective view of high data rate link based on graphene transmitter (Tx) and receiver (Rx). (**b**) Fabricated chip on a 70-μm thick SiC (chip size: 15 × 15 mm^2^) consists of frequency mixer ICs (left, circuit size: 1.35 × 1.1 mm^2^) and integrated power detector ICs (right, circuit size: 1.35 × 0.7 mm^2^).

Figure 1a shows a prospective application for graphene based high speed electronics. It is a point-to-point high capacity wireless link operating at 90 GHz atmospheric window. Simplified block diagrams of a typical transmitter and receiver are also shown. The basic function of a transmitter is to modulate the data into a high frequency sine wave carrier signal that can be radiated by an antenna. The information can be mapped into either amplitude, phase or frequency of the carrier signal by a modulator. To use the RF spectrum more efficiently the carrier of the modulated signal need to be converted to higher frequencies. This can be done by frequency mixers. To provide frequency conversion, an external sinusoidal source called local oscillator (LO) is needed. Generally, it is difficult to generate such a signal with sufficient power and signal stability at mm-wave frequencies. Therefore, there is need for a frequency multiplier, which generates an output signal whose output frequency is a multiple of its input frequency. Finally, an amplifier can be used to increase the output power of the transmitter for compensating different losses between the transmitter and the receiver. The function of a receiver is to recover the data by more or less reversing the function of the transmitter components. Hence, the receiver consists of essentially the same RF components.

To achieve complete wireless communication systems based on graphene it is needed to realize all the above mentioned components with graphene technology. Transistors are versatile elements and by choosing proper operating points and passive components, all needed functionalities are attainable.

Therefore, G-FETs are the only needed graphene based active elements for these circuits. In addition, it is desirable to have whole transmitter and receiver chain on a single chip which facilitates the very broadband performance needed for the high data rate applications. Hence, developing G-FET based MMICs is a key requirement to obtain graphene based high speed wireless systems.

**Figure 2:**
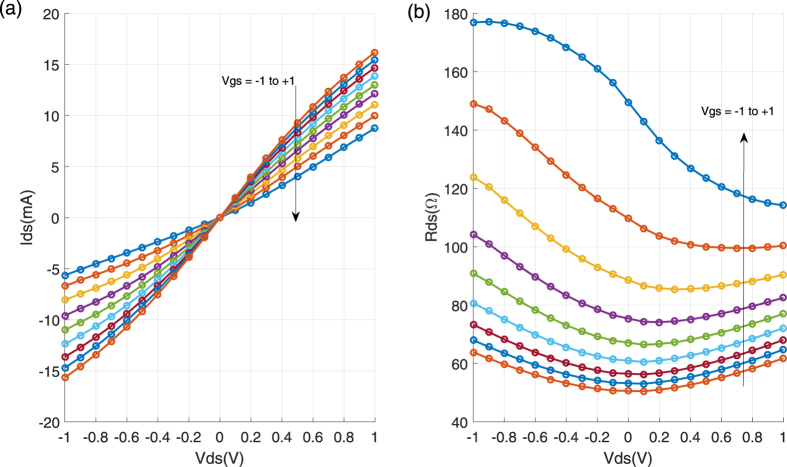
G-FET DC characteristic. (**a**) Output characteristics of the G-FET with Vgs ranging from −1 to 1 V with step of 0.25 V. (**b**) Drain-source resistance versus drain bias.

Circuit performance depends on both device and circuit technology. Device (G-FET) technology has recently shown a rapid progress. However, to have G-FET based amplifiers operating at 90 GHz band, G-FETs with f_MAX_ more than 200 GHz is needed. Therefore, more development in G-FET technology is needed to realize G-FET based amplifiers at this frequency band. However, for other applications where the G-FETs operate in other modes, the existing G-FETs technology based on hydrogen intercalated graphene is sufficient. This is because this type of graphene exhibits a high carrier mobility with a very low sheet resistance. It results in a reduction of channel resistance and consequently achieving small time constants in G-FETs needed for higher frequency operations. Developing microstrip MMIC process on SiC substrates is a laborious task especially at high frequencies where the parasitic elements and unwanted propagation modes can significantly deteriorate the circuit performance. In addition, graphene compatibility is extremely important and because of that many existing circuit fabrication technologies cannot be directly applied for the development of graphene based MMICs. These process have several steps that can potentially degrade graphene. One way to avoid this problem is to fabricated the passive elements first and then transfer graphene and perform G-FETs fabrication^24^. However, this method cannot be used for epitaxial graphene since the graphene already exists on the SiC substrate. Based on a Gallium Nitride HEMT MMIC process^27^, we have developed a unique fabrication process technology that preserve graphene property after more than 30 process steps. Graphene is very susceptible to plasma processes and therefore it should be encapsulated. This can be performed by utilising a thick (90 nm) layer of Al_2_O_3_ deposited by the atomic layer deposition (ALD) method^28^. A thick layer of ALD Al_2_O_3_ exhibits a poor thermal conductance and a high tensile stress. Since SiC via hole etching is performed at very high powers, the generated heat causes the Al_2_O_3_ layer to crack. This completely damages G-FETs as well. To avoid it, a thin layer (15 nm) of ALD Al_2_O_3_ is deposited and it is followed by 100 nm SiN sputtered at a very low density plasma. This process does not damage the graphene. In addition, the new encapsulated layer can completely protect graphene from the remaining plasma steps. Furthermore, the new layer has a better thermal conductance and a lower stress and can survive the via hole etching process. In this process the SiC substrate is thinned down to 70-μm providing a platform to develop circuits up to 250 GHz. By using our device and circuit technologies we are able to realize needed RF modules except amplifiers at 90 GHz band. RF signal in mm-wave can be generated by using G-FET based frequency multipliers. However, since the output power of these frequency multipliers are very low^5^,^6^,^7^,^8^ amplifiers are needed to achieve practical LO sources. The fabricated MMIC and the corresponding circuits are shown in Fig. 1b. In this figure, the left circuit is a single-ended resistive mixer that can be used for the signal modulation and demodulation as well as frequency conversions. The passive part of the mixer consists of RF, LO and IF filters as well as an integrated bias tee to bias the gate of the transistor. In addition, a matching network is used for matching the LO port. The RF signal is applied to the drain of the G-FET and the IF signal is extracted from the same terminal. The right circuit is an integrated power detector that can be used for signal demodulation. The same passive elements are used. The RF signal is applied to the gate of the G-FET and the rectified signal is extracted from the drain terminal. The I-V characteristic and drain-source resistance of the fabricated 250-nm G-FETs are plotted in Fig. 2 showing that the channel resistance is almost constant around zero drain bias over a wide range of gate voltage. This property allows the realization of very linear mixers and power detectors, needed for high data rate communications.

### MMIC performance in high data rate applications

**Figure 3:**
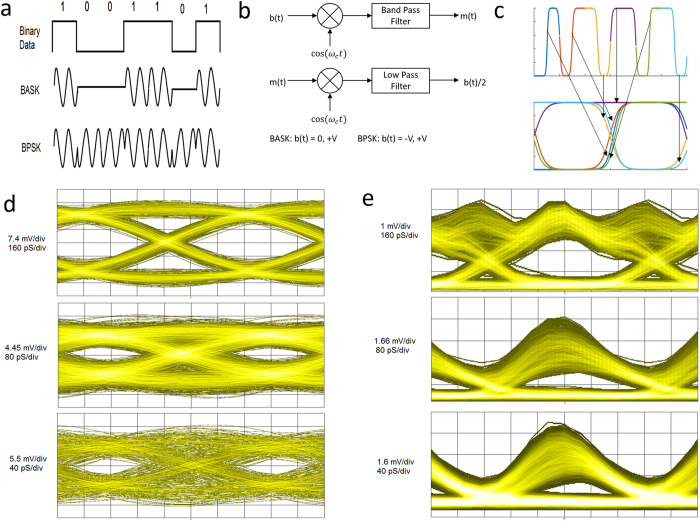
Signal modulation and eye diagram. (**a**) Binary amplitude and phase digital modulation. (**b**) BASK and BPSK signal modulation and demodulation by using a mixer. (**c**) Formation of an eye diagram by using two consecutive bits. (**d,e**) Eye diagrams formed by demodulation of 90 GHz BPSK (**d**) and BASK (**e**) signals respectively at the rate of 1, 2 and 4 Gbps using G-FET MMICs.

The most fundamental digital modulation techniques are binary amplitude-shift keying (BASK) and binary phase-shift keying (BPSK) where binary information is coded in the amplitude and phase of the carrier signal respectively (Fig. 3a). A mixer can be used for modulation and demodulation of the BASK and BPSK signals as shown in Fig. 3b. In this figure b(t) and m(t) are the binary data and modulated signal respectively. For BASK the binary data should be represented by unipolar voltages (0, +V) while for BPSK it is bipolar voltages (−V, +V). BPSK modulation is less susceptible to the noise than BASK modulation, since the amplitude of the carrier is more affected by the noise than its phase. The signal quality in high speed communication systems are mainly evaluated and analysed by a special type of diagram, called an eye diagram. The eye diagram is formed by overlapping the trace of a certain number of bits in time domain. Due to the bandwidth limitation and device instability, the transitions do not line up on top of each other completely and an eye-shaped pattern emerges^29^ (Fig. 3c). Much information can be obtained from the eye diagram such as the amount of noise that can be tolerated, the amount of signal distortion and sensitivity to the timing error. Differences in amplitude and timing of bits can cause the eye opening to shrink and degrade data transmission quality. A BPSK modulated signal at 90 GHz is used to assess the performance of the G-FET demodulator MMIC. The input signal is modulated at the rate of 1, 2 and 4 Gbps. The eye diagrams of the demodulated data are shown in Fig. 3d. As expected, the opening of the eye diagram shrinks with increasing data rate. This is because at higher data rates the data quality is more affected by distortion and bit timing error (jitter). The signal-to-noise ratio (SNR) and noise margin of a 1 Gbps demodulated signal are 11 dB and 17 mV respectively. For 2 and 4 Gbps signals, the SNR and noise margin are reduced to 6–7 dB and 5 mV. In addition, the jitter is estimated to be 160–180 ps. This limits the data rate to about 5 Gbps at which the eyes become completely closed. More details of the mixer performance can be found in the Supplementary information. As mentioned before, a BASK signal is more susceptible to noise. In addition, it has a lower power efficiency since it does not have a constant envelope. However, it offers a possibility of having a simple demodulator circuit. The circuit is based on a power detector and a low pass signal (Fig. 1b right). This topology does not need a LO signal which makes it to be more compact and cost effective. G-FETs can be used as a power detector in mm-waves and THz region for signal rectification and detection.

To evaluate the performance of the power detector based demodulator MMIC, a BASK signal at 90 GHz with modulation rates of 1, 2 and 4 Gbps is used. Due to the band width limitation of our arbitrary waveform generator, the BASK signals have triangular-shaped pulses (Supplementary information). The eye diagrams of demodulated signals are presented in Fig. 3e. As can be seen, the relative amplitude variation is larger compared to BPSK especially at high data rates. In this case, the noise margin is about 1–2 mV. The extracted SNR are approximately 8, 6 and 5 dB respectively and the jitter is 160–180 ps. Therefore, the maximum data rate that can be demodulated is below 5 Gbps.

The performance of the demonstrated G-FET based demodulators and this work is shown in Table 1. In the Supplementary information, details of the power detector performance are presented.

**Figure 4:**
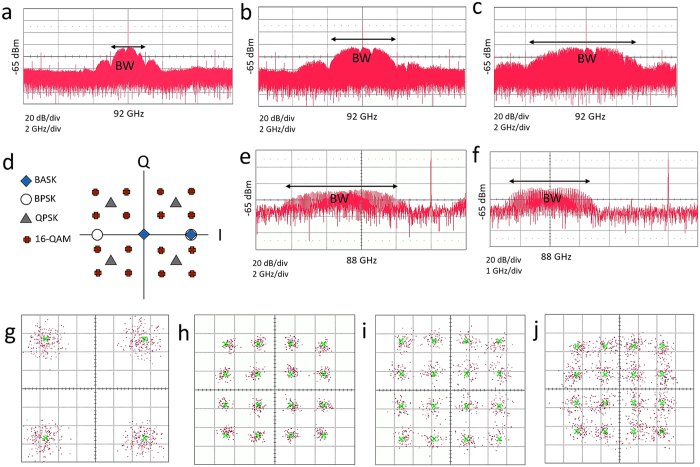
Spectrum and constellation of modulated signals. (**a–c**) Spectrum of BPSK modulated signal at the rate of 1, 2 and 4 Gbps respectively. (**d**) Constellation diagram for BASK, BPSK, QPSK and 16-QAM modulations. (**e,f**) Spectrum of up-converted 8 Gbps QPSK and 16-QAM signals. (**g**) Constellation diagram for 8 Gbps QPSK signal. (**h,i,j**) Constellation diagram for 4, 8 and 16 Gbps 16-QAM signals respectively. The green crosses are the expected values and the purple dots are the measured points.

The MMIC mixer circuit can be used for signal modulation as well. As shown in Fig. 3b, a BPSK signal is generated by mixing a bipolar base band signal (±V) and a carrier signal. The spectrum of the generated BPSK signal at the rate of 1, 2 and 4 Gbps are plotted in Fig. 4a, b and c respectively. The carrier frequency is 92 GHz and the generated signal exhibits a SNR above 20 dB. As expected for the BPSK signal, the null-to-null bandwidth is about twice the data rate. In addition, due to the signal leakage, the spectrum also contains the LO signal. This can be mitigated by utilizing more complex modulator circuits^29^,^30^. In order to use the available RF bandwidth more efficiently it is needed to utilize more complex modulation schemes. In these modulation schemes more information can be embedded into the amplitude and phase of the carrier signal. The in-phase (I) and quadrature (Q) components of transmitted symbols can be visualized as points on a constellation diagram (Fig. 4d). Quadrature phase-shift keying (QPSK) and 16-quadrature amplitude modulation (16-QAM) have 4 and 16 symbols representing 2 and 4 bits per symbol respectively. Generally, it is difficult to carry out these types of modulations directly at mm-wave frequencies. Hence, the signal modulation is performed at low frequencies and by using a mixer, the carrier frequency is up-converted to the desired frequencies. To keep the modulation constellation intact, it is necessary to have a highly linear mixer. As shown in Fig. 2b, the channel resistance of the G-FET is almost constant over a wide range of gate voltages. This suggests that G-FET based mixer will exhibit a high linearity. To evaluate the performance of the mixer, QPSK and 16-QAM modulated signals at 4 GHz are used. The 8 Gbps QPSK modulated signal is up-converted to 88 GHz by using the MMIC mixer. The spectrum of the signal is plotted in Fig. 4e. The data is retrieved from the lower sideband and the corresponding constellation is shown in Fig 4g. From this diagram a BER of 10^−6^ can be estimated. The experiment is repeated by 16-QAM signals modulated at the rate of 4, 8 and 16 Gbps. The reconstructed constellation diagrams are plotted in Fig. 4h, i and j and the extracted BERs are about 10^−6^, 10^−5^ and 10^−4^ respectively. It can be seen that the achieved constellation diagrams are not distorted meaning that the mixer is highly linear. Fig 4f shows the spectrum of the 16-QAM signal modulated at the rate of 8 Gbps. It is seen that the required bandwidth is about half of the QPSK modulation with the same data rate as expected. Table 2 summarizes the performance of multi-Gbps modulators in other technologies (at 70–90 GHz) as well as our results.

## Discussion

To achieve high frequency devices base on graphene having a high carrier mobility is not sufficient. Graphene films should have high carrier density as well in order to obtain low sheet resistance levels needed for high frequency applications. These properties exist in hydrogen intercalated epitaxial graphene and thereby it is more beneficial for RF devices and circuits. The development of G-FET MMICs that can perform multi-Gbps signal modulation and demodulation at mm-wave makes a start on the realization of complete high data rate transceivers based on graphene. Prior to this work, the operating frequency and data rate of the demonstrated graphene based ICs are far below the performance of the existing technologies. This is mainly due to the lack of high quality process technology and material. For high data rate communication, the linearity of the RF components plays a key role. The fabricated MMIC mixer is highly linear allowing to generate and retrieve multi-Gbps data at 90 GHz atmospheric window. This work demonstrates G-FETs’ great potential for high data rate communication systems. To achieve complete wireless communication systems based on graphene, more development in G-FET technology is needed to realize amplifiers at mm-wave band.

## Methods

### Growth of epitaxial graphene

Epitaxial graphene is grown at 1600 °C and 30 mbar pressure by CV technique on (15 × 15) mm^2^ nominally on-axis 4H-SiC (0001) or 6H-SiC (0001), Si-face semi-insulating chemo-mechanically polished substrates. Graphene layers are grown under an argon laminar flow at hot-wall Aixtron VP508 and Aixtron G5 reactors where graphene growth on 4″ SiC substrates is under development. The process depends critically on the creation of the flow conditions in the reactor that control Si sublimation rate and enable mass transport of hydrocarbon to the SiC surface. Reynolds number (Re) measures the ratio of inertial forces to viscous forces and consequently quantifies the relative importance of these two forces in a given gas flow. Tuning the value of the Re number allows to form a thick enough Ar boundary layer to prevent Si sublimation. This lets the diffusion of hydrocarbon to the SiC surface and growth of epitaxial graphene. The intercalation of hydrogen is obtained *in situ* at the temperature of 1100 °C and 900 mbar pressure. In our samples the presence of hydrogen atoms is maintained up to 700 °C, high enough to meet the requirements of high-speed electronics and high-temperature sensing.

### MMIC fabrication

The fabrication process has about 30 different steps including 3 ebeam lithography and 13 photolithography steps. Ebeam-lithography is used for the fabrication of G-FETs since ebeam resists are more compatible with graphene. For G-FET passivation a combination of ALD Al_2_O_3_ (15 nm) and sputtered SiN (100 nm) layers are utilized. The rest of the fabrication process are based on photolithography. For forming thin film resistors, tantalum Nitride (TaN) is sputtered. The metal-insulator-metal (MIM) capacitors are based on SiN. After the fabrication of MIM capacitors, dielectric via etching is performed to reach to the G-FET metal pads for the deposition of the large pads and transmission lines. Gold electroplating is done to reduce the metal resistivity and form air-bridges. Then a thick layer of resist is used to protect the surface and the chip is glowed to a carrier wafer for the back side processing. The substrate is thinned down to 70 μm and polished by a CMP process. For via hole formation, a thick layer of nickel is plated as a hard mask and high power ICP is used to etch via holes. The nickel mask is wet striped and a seed layer is sputtered on the back side of the chip. Finally, gold is plated for the metallization of the ground plane and via holes.

### MMIC measurement

An Agilent signal generator and an OML S10MS W-band source module are used to provide the LO signal. The digital modulated signals at baseband and intermediate frequency are generated by a Keysight M8195A 65-Gsps sampling rate 8-bit arbitrary waveform generator. The millimeter wave output is measured by a Lecroy Labmaster 10-100Zi real time oscilloscope which has 100 GHz input bandwidth and 240 Gsps sampling rate. High data rate BASK (on off keying) signal is generated by an in-house fabricated W-band frequency multiplier module based on InP DHBT MMIC. The data captured by the real time oscilloscope is processed using Matlab code.

## Additional Information

**How to cite this article**: Habibpour, O. *et al*. Wafer scale millimeter-wave integrated circuits based on epitaxial graphene in high data rate communication. *Sci. Rep.* **7**, 41828; doi: 10.1038/srep41828 (2017).

**Publisher's note:** Springer Nature remains neutral with regard to jurisdictional claims in published maps and institutional affiliations.

## Supplementary Material

Supplementary Information

## Figures and Tables

**Table 1:** Comparison of G-FET based demodulators performance.

Reference	Gate length (μm)	Carrier mobility (cm^2^/Vs)	Sheet resistance (Ω/Vs)	Operation Mode	Data rate	Operation Frequency (GHz)
18	1	900–1000	700–800	Direct detection	20 Mbps	5
This work	0.25	3500–6500	150–250	Direct detection	4 Gbps	90
24	0.9	1000–3000	1000–2000	Heterodyne	20 Mbps	5
This work	0.25	3500–6500	150–250	Heterodyne	4 Gbps	90

**Table 2:** Performance comparison of the G-FET MMIC modulator with existing technologies at 70–90 GHz (BER < 10^−5^).

Reference	Frequency (GHz)	Modulation	Data rate (Gbps)	Technology
31	70–80	16 QAM	10	100 nm GaAs p-HEMT
32	70–80	QPSK	18	130 nm SiG BiCMOS
33	83–88	BPSK/QPSK	2.5	65 nm CMOS
34	92–95	16 QAM	6	100 nm GaAs p-HEMT
This work	80–90	16 QAM	8	250 nm G-FET
