# Supplementary material for: Wafer scale millimeter-wave integrated circuits based on epitaxial graphene in high data rate communication
Source: Sci Rep. 2017 Feb 1;7:41828. doi: 10.1038/srep41828 (PMC5286424; doi:10.1038/srep41828)
Supplement: Supplementary Information [file srep41828-s1.doc]

**Supplementary information**

Wafer scale millimeter-wave integrated circuits based on epitaxial graphene in high data rate communication

Omid Habibpour, Zhongxia Simon He, Wlodek Strupinski, Niklas Rorsman and Herbert Zirath

**S1-Epitaxial graphene characterization**

Raman spectroscopy and mapping are utilized to investigate the graphene layer structure by using a Nd:YAG laser (532nm). The laser spot size is about 300 nm with average laser power below 0.1mW. All spectra are corrected by substrate subtraction procedure and Lorentzian shape fitting. Figure S1 shows the G peak intensity as well as 2D peak shift Raman map. The map demonstrates the SiC surface morphology including terraces and step edges. The narrow FWHM of 35 cm^–1^ and the single Lorentzian fitting suggests that the area is mainly monolayer graphene. On terrace edges, the intensity of the G peak increases and the 2D peak broadens (FWHM=62 cm^–1^).

**Figure S1**. Raman measurements results of graphene on SiC a) spectrum, b) map 20 mm x 20mm (2LG on the step edges is depicted with red), c) histogram for FWHM.

Hall structures are used to measure the carrier mobility. The area located entirely on a single trace exhibits carrier mobility exceeding 7000cm^2^/Vs. However, the area covering 2-3 terraces exhibits mobility in the range of 3500–5000cm^2^/Vs. Hence developing graphene with large area terraces paves the way for achieving devices with better performance.

**S2-Power detector MMIC**

The open circuit (R_V_) and short circuit (R_I_) responsivity of the MMIC power detector is shown in Fig. S2. As can be seen R_V_ ≈ 7-10 V/W and R_I_ ≈ 75-100 mA/W. For high data rate application, the load termination should be 50 Ω since the open or short circuit terminations limit the band width. Figure S3 presents measured DC output voltage versus RF input at 98 GHz with 50 Ω termination. It is seen the detector is exceptionally linear over a wide range of input signal and the responsivity is R_V,50Ω_ = 5 V/W. In order to absorb the input signal completely and also avoid distortion in the detected signal, the RF port should be matched over the signal bandwidth. In a G-FET power detector, the RF signal is applied to the gate of the G-FET. Hence the gate of the G-FET should be matched. The measured S-parameter is shown in Fig. S4. It is seen that around 90 GHz the port is matched and hence we use this frequency for high data rate signal detection.


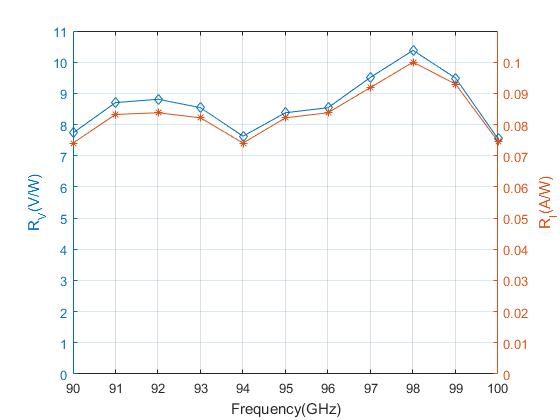


**Figure S2.** The responsivity of the power detector MMIC


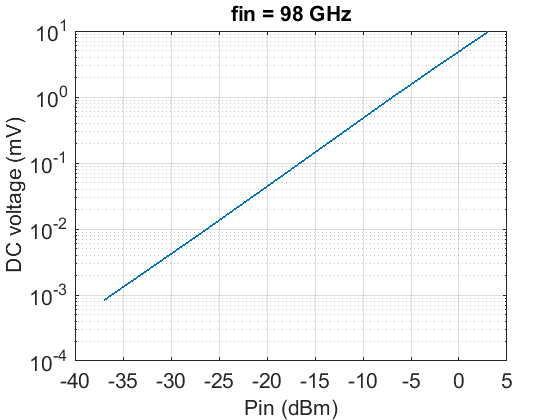


**Figure S3.** The output voltage vs input power at 98 GHz with a 50-Ω load


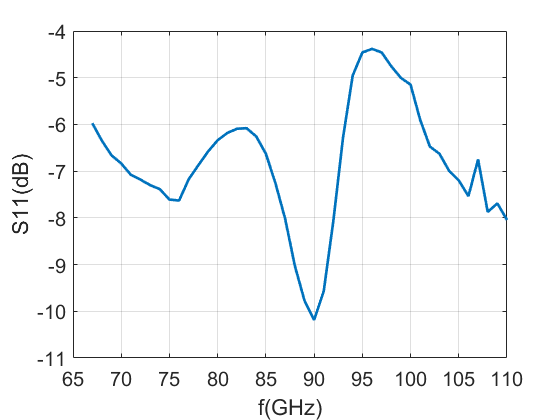


**Figure S4.** Measured return loss of the MMIC power detector

**S3-Mixer MMIC**

The mixer 1^st^ and 3^rd^ response at 90 GHz is shown in Fig. S5. The extracted conversion loss is about 18-19 dB and input 3^rd^ order interception point (IIP3) is estimated to be 15-16 dBm. In addition, RF and LO return losses versus frequency is shown in Fig. S6. It can be seen that the RF port is matched over a wide frequency range (85-110 GHz), however, the LO port is only matched around 90 GHz.


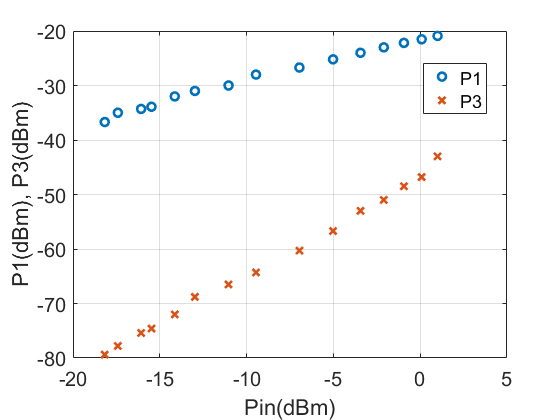


**Figure S5.** Measured 1^st^ and 3^rd^ order response of the MMIC mixer


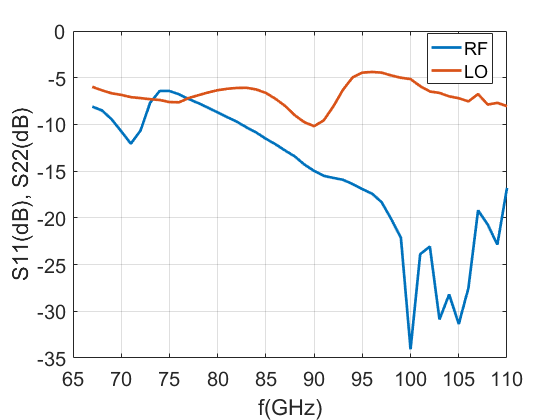


**Figure S6.** Measured RF and LO return loss of the MMIC mixer

**S4-BASK signal generation**

Generating a high data rate BASK signal at high frequencies is a challenging task. By using an arbitrary waveform generator (M8195A 65-GSps), we can generate a BASK signal at 30 GHz. Since the sampling rate is 65 GSps, the frequency component of the generated waveform is limited to 32.5 GHz and above that is omitted. A 4 Gbps data with rectangular pulse shape at 30 GHz has frequency components exceeding 32.5 GHz which are truncated. Hence, the generated signal has triangular-shaped pulses as shown in Fig. S7.


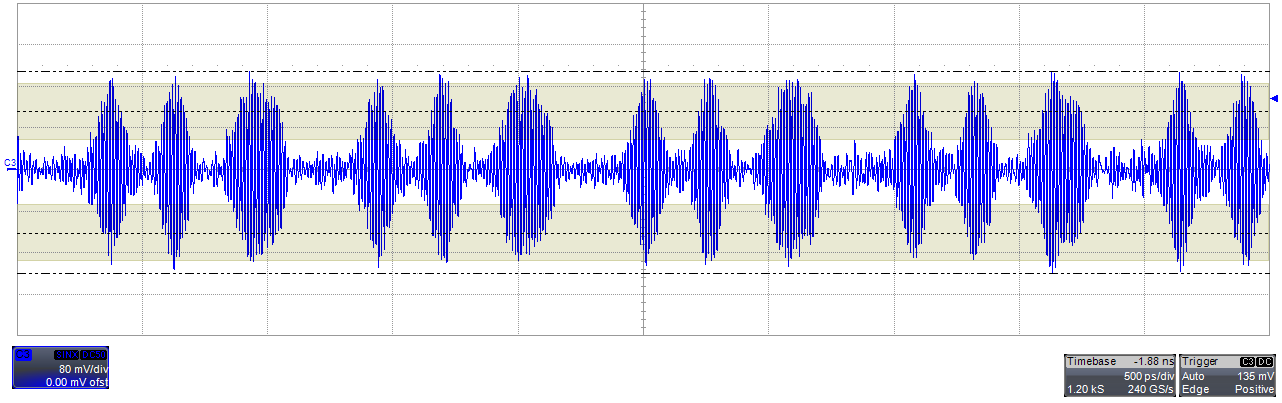


**Figure S7.** 4 Gbps BASK signal generated by an Arbitrary waveform generator and a frequency tripler at 90 GHz
